# Characteristics of Randomized Controlled Trials Designed for Elderly: A Systematic Review

**DOI:** 10.1371/journal.pone.0126709

**Published:** 2015-05-15

**Authors:** Karen Broekhuizen, Axel Pothof, Anton J. M. de Craen, Simon P. Mooijaart

**Affiliations:** 1 Department of Gerontology and Geriatrics, Leiden University Medical Center, Leiden, Netherlands; 2 Institute for Evidence-Based Medicine in Old Age, IEMO, Leiden, Netherlands; University Hospital Oldenburg, GERMANY

## Abstract

**Objectives:**

To quantify the proportion of randomized controlled trials (RCTs) specifically designed for elderly, and to assess their characteristics, as compared to RCTs not specifically designed for elderly.

**Design:**

Review and synthesis of published literature.

**Measurements:**

We searched PubMed for articles published in the year 2012. We included RCTs. Articles were excluded if not conducted with human subjects or if findings of secondary analyses were reported. A random sample of 10% was drawn and of this selection the following trial characteristics were extracted: sample size, disease category, age of sample, and age-related inclusion criteria. Clinical trials were defined to be specifically designed for elderly if a lower age cut-off of ≥ 55 years was used, or when participants had an average age of ≥ 70 years.

**Results:**

The search strategy yielded 26,740 articles, from which a random sample was drawn, resulting in 2375 articles. After exclusion, data was extracted from 1369 publications. Of these 1369 RCTs, 96 (7%) were specifically designed for elderly. In comparison with trials not designed for older adults, trials designed for elderly contained a significantly larger median number of participants (125 vs. 80, p = 0.008) significantly more trials designed for elderly fell into the disease categories eye (6% vs. 2%, p = 0.005), musculoskeletal (13% vs. 7%, p = 0.023) and circulatory system (16% vs. 9%, p = 0.039). No significant difference was observed with regard to the other disease categories.

**Conclusion:**

There is a low proportion of RCTs specifically designed for elderly. As older patients will increasingly form the majority in medical practice, there is an urgent need for stronger evidence for the formulation of treatment guidelines specifically for older adults.

## Introduction

Randomized controlled trials (RCT) are the hallmark of evidence-based medicine and form the basis of treatment guidelines. Elderly form the majority of patients in medical practice and should therefore be sufficiently represented in randomized clinical trials. [1–4] As physical, psychological and social functioning of older patients are substantially different from younger patients, studies specifically designed for elderly are warranted. Since older patients often present with comorbidities and/or polypharmacy, the balance between pros and cons of a specific treatment might be systematically different. Therefore, clinical trials enrolling older patients may require a more specific approach.

Earlier studies have signalled an underrepresentation of older adults in disease-specific clinical trials, e.g. in trials on the treatment of cardiovascular diseases [5–9] and in clinical trials on cancer treatment. [10–12] Overall, no studies have given a total overview of the number of RCTs specifically designed for elderly, nor what the characteristics of these trials are.

We performed a systematic review to assess the proportion of RCTs specifically designed for elderly, and to assess their characteristics, as compared to RCTs not specifically designed for elderly.

## Methods

### Search strategies

A systematic search was conducted to identify RCTs that were published in 2012. The following search string was used in the PubMed database: ("Randomized Controlled Trial"[Publication Type] OR rct[ti] OR randomized[ti] OR randomised[ti]) AND ("2012/01/01"[PDAT]: "2013/01/01"[PDAT]). A systematic review protocol was developed and is available on request.

### Study selection

A random sample of 10% was drawn from the results of the search strategy (by using IBM SPSS Statistics version 20.0). First, title and abstract of the publications of this sample were unduplicated and reviewed. Publications were included if written in English and reporting a study with RCT design. Publications were excluded if reporting a study protocol, pilot study or systematic review. Further, publications reporting studies in non-human subjects or reporting a secondary analyses, e.g. process evaluations, results from post-hoc analyses, were excluded. If studies met the inclusion criteria, or if uncertainty about eligibility remained, full texts were retrieved.

### Data extraction

From full texts, the following characteristics were extracted: sample size, disease category, age of study sample, and age-related inclusion criteria. Disease category was classified according to the International Classification of Diseases (ICD-10) of the World Health Organization (WHO). This classification system includes the analysis of the general health status of population groups. Trials were classified in 22 categories based on the disease of the trial participants or based on the primary endpoint (if participants were healthy subjects or if participants were not selected for participation based on their disease). The researcher performing the classification followed an online training provided by the WHO website. [13]

From the total sample the number of RCTs specifically designed for elderly was determined. In order to label trials as specifically designed for elderly, the trial participants had to be included according to an age-related cut-off value or the average age of the participants had to exceed an age-related cut-off value. We chose to label a trial as specifically designed for elderly if trial participants were aged ≥ 55 years as defined by a lower age cut-off, or with an average age of ≥ 70 years. As these cut-off values are arbitrary, as a sensitivity analysis, we also determined the quantity of RCTs specifically designed for elderly according to other cut-off values: 1) if participants were aged ≥ 65 years, or with an average age of ≥ 75 years, 2) if participants were aged ≥ 65 years, and 3) if participants were aged ≥ 75 years.

One researcher (AP) performed study selection and data extraction of all publications. In case of uncertainty, a second researcher (KB) was consulted. Study selection and data extraction of 10% of the publications was done twice by two researchers (AP and KB) in order to maximize inter-rater agreement and ensure a solid categorization procedure. The two researchers reached consensus on the procedure for the remaining articles. Authors were contacted in case of incomplete data in publications.

### Statistical analysis

Measures of central tendency of continuous variables from the trials were recorded as mean (SD) or median (IQR). For dichotomous variables the number of subjects with the characteristic divided by the total number of subjects was recorded.

Trial characteristics of RCTs specifically designed for elderly were compared to characteristics of RCTs that were not designed for older adults, using the Mann-Whitney U test. Since it was our intention to describe the characteristics of trials independent of sample size, all analyses were unweighted. All analyses were performed with IBM SPSS Statistics version 22.0. Statistical significance was accepted at P<0.05.

## Results

### Results from search strategy

**Fig 1:**
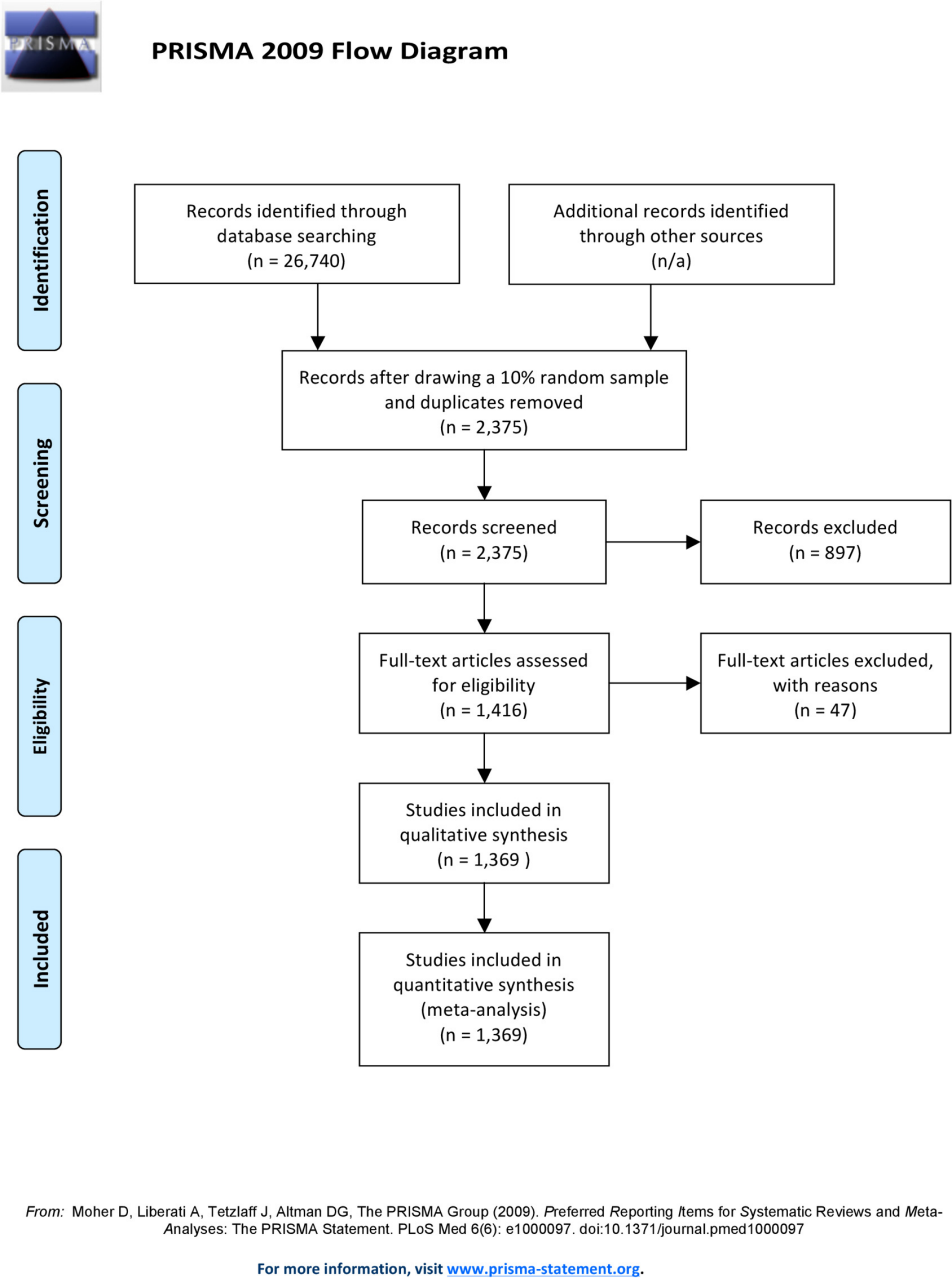
Flow chart for inclusion of studies. PRISMA flow chart of the results from the performed search strategy and selection process.

The search strategy yielded 26,740 publications. We took a random selection of 10%, which resulted in 2375 publications. After screening titles and abstracts and deleting duplicates, 1416 publications remained (Fig 1). Most studies were excluded because they did not have a RCT design (n = 319), contained secondary analyses (n = 152) or were performed in non-humans (n = 148). Of the 1416 remaining publications, we were able to retrieve 1369 (97%) in fulltext format.

### Number of RCTs specifically designed for elderly

**Fig 2:**
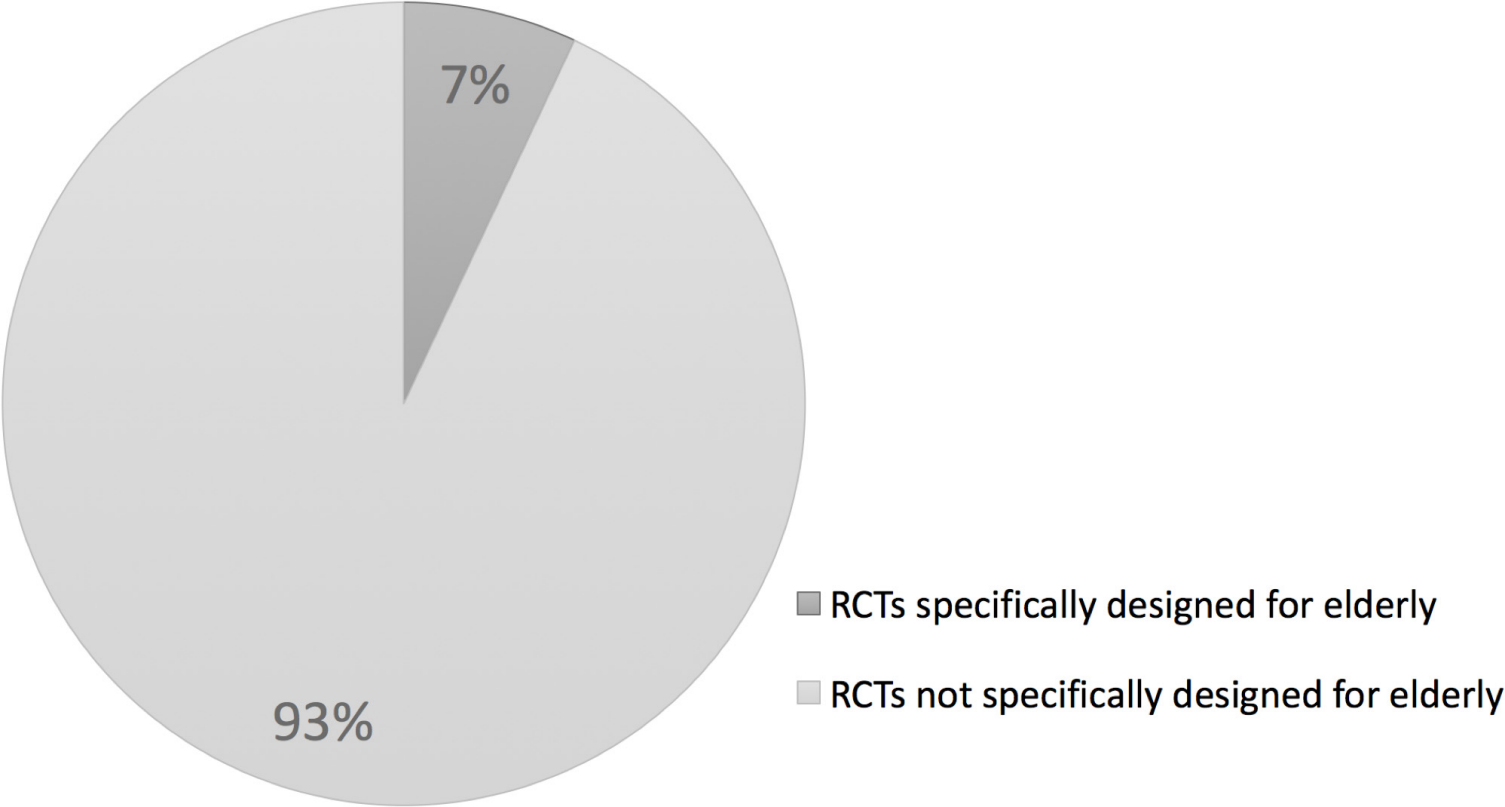
The representation of elderly in 1369 randomized controlled trials performed in 2012. Pie chart showing the amount and proportion of randomized controlled trials designed for elderly.

A total of 96 (7%) trials of the 1369 trials were specifically designed for elderly according to our age-related cut-off values if trial participants were aged ≥ 55 years, or with an average age of ≥ 70 years (Fig 2). Sensitivity analyses showed that, when applying more stringent cut-off values (if participants were aged ≥ 65 years, or with an average age of ≥ 75 years), 41 (3%) of the trials was designed for elderly. When applying one-sided cut-off values (participant age is ≥ 65 and ≥ 75 years respectively), 27 (2%) and 2 (0.1%) of the trials was designed for elderly.

### Trial characteristics

In S1 Appendix, a full database of all 1369 included publications, including authors/titles and trial characteristics can be accessed. A description of the main trial characteristics of the 1369 trials included in this review can be found in Table 1. Median age of the trial participants was 46 (IQR 29–60) years. An age-related lower cut-off value was used by 787 (58%) studies with a median age of 18 (IQR 18–20) years. The median number of participants per trial was 82 (IQR 40–215). As can be seen in Table 2, most trials were classified into the following 3 disease categories: behavioural (N = 190; 14%), circulatory (N = 132; 10%) and metabolic diseases (N = 131; 10%). Of all 1369 trials, 5–10% were classified into categories neoplasms, nervous system, musculoskeletal diseases, infectious diseases, digestive diseases, genitourinary diseases and respiratory diseases. Less than 5% of the trials were classified into other categories.

**Table 1:** Main trials characteristics of 1369 included RCTs.

Main trial characteristics				
	All included trials	Trials specifically designed for elderly^1^	Trials not specifically designed for elderly	p^3^
	N = 1369	N = 96	N = 1273	
Number of participants, N (median, IQR^2^)	82 (40–215)	125 (48–316)	80 (40–210)	0.008
Age of participants, years (median, IQR)*	46 (29–60)	73 (71–77)	44 (28–57)	0.000
Lower cut-off value for age, years (median, IQR)**	18 (18–20)	65 (49–65)	18 (18–18)	0.000

* Data are based on 1240 (91%) trials

** Data are based on 787 (57%) trials

^1^ If trial participants were aged ≥ 55 years, or with an average age of ≥ 70 years

^2^ Interquartile range, difference between 25^th^ and 75^th^ percentile is reported

^3^ P for difference between trials specifically designed for elderly and trials not specifically designed for elderly, tested with an Independent-Samples Mann-Whitney U test

**Table 2:** Disease categories of 1369 included RCTs.

Disease category (N, %)				
	All included trials	Trials specifically designed for elderly	Trials not specifically designed for elderly	P^1^
	N = 1369	N = 96	N = 1273	
Infectious	77 (6%)	5 (5%)	72 (6%)	0.854
Neoplasms	117 (9%)	11 (11%)	106 (8%)	0.290
Blood	17 (1%)	0 (0%)	17 (1%)	0.255
Metabolic	131 (10%)	4 (4%)	127 (10%)	0.062
Behavioral	190 (14%)	10 (10%)	180 (14%)	0.309
Nervous system	111 (8%)	11 (11%)	100 (8%)	0.212
Eye	30 (2%)	6 (6%)	24 (2%)	*0*.*005*
Ear	4 (0%)	0 (0%)	4 (0%)	0.582
Circulatory	132 (10%)	15 (16%)	117 (9%)	*0*.*039*
Respiratory	68 (5%)	5 (5%)	63 (5%)	0.910
Digestive	80 (6%)	4 (4%)	76 (6%)	0.468
Skin	34 (3%)	1 (1%)	33 (3%)	0.346
Musculoskeletal	104 (8%)	13 (13%)	91 (7%)	*0*.*023*
Genitourinary	74 (5%)	4 (4%)	70 (6%)	0.578
Pregnancy	25 (2%)	0 (0%)	25 (2%)	0.166
Perinatal	18 (1%)	0 (0%)	18 (1%)	0.241
Congenital	3 (0%)	0 (0%)	3 (0%)	0.634
Symptoms	7 (1%)	0 (0%)	7 (1%)	0.466
Injury	15 (1%)	1 (1%)	14 (1%)	0.958
External	9 (1%)	0 (0%)	9 (%)	0.408
Contact with health services	35 (3%)	1 (1%)	34 (3%)	0.329
Codes for special purposes/other	88 (6%)	5 (5%)	83 (7%)	0.613

^1^ Pearson’s Chi square test

### Comparison of trial characteristics

From Table 1 can be seen that RCTs designed for elderly contained significantly more participants in comparison with trials not designed for older adults (p = 0.008); medians were 80 (IQR 40–210) and 125 (IQR 48–316) respectively. Significantly more trials designed for elderly fell into the disease categories eye (6% vs. 2%, p = 0.005), musculoskeletal (13% vs. 7%, p = 0.023) and circulatory system (16% vs. 9%, p = 0.039), compared to trials not designed for older adults. No significant difference was observed with regard to the other ICD-10 disease categories.

Differences were no longer significant when we repeated this comparative analysis based on more stringent cut-off values (if participants were aged ≥ 65 years, or with an average age of ≥ 75 years) and on one-sided cut-off values (participant age is ≥ 65 and ≥ 75 years respectively).

## Discussion

This review has two main findings. First, RCTs designed for elderly account for 7% of all performed RCTs in 2012. Second, trial characteristics of RCTs designed for elderly compared to RCTs not designed for older adults are different with respect to the amount of participants and their distribution across disease categories.

The results in this review reinforce previous findings. So far, two studies have examined the underrepresentation of elderly in clinical trials using a similar approach, by reporting the proportion of clinical trials specifically designed for elderly. In one systematic review it was reported that only 3 (2%) of the 155 RCTs on four widely prescribed drugs were exclusively designed for patients aged 65 years and older. [14] Another systematic review found that only 2 (7%) of the 59 chronic heart failure trials published from 1985 to 1999 exclusively enrolled participants aged 65 years and over. [9] According to the results in our review, 2% of the RCTs in 2012 was designed for elderly aged 65 and over, which is comparable and even lower the above-mentioned proportions.

Our results also show significant differences in trial characteristics between RCTs designed for elderly and not specifically designed for elderly. Firstly, RCTs designed for elderly were consistently larger with respect to the amount of participants, in comparison to trials not designed for older adults. As post-hoc examination of our data show that 2 of the 3 RCTs that enrolled over 10,000 participants were large prostate cancer screening trials, an explanation could be that in RCTs specifically designed for elderly a different type of treatment is investigated, requiring a larger amount of participants. As we did not extract any data regarding the type of treatment, this reason remains speculative. Secondly, findings of our review show a significantly higher proportion of RCTs designed for elderly regarding eye, circulatory and musculoskeletal diseases. To date, no other study particularly compared trial characteristics of RCTs specifically designed for elderly with RCTs not designed for older adults, but it is likely that these reflect the relatively high prevalence of eye, circulatory and musculoskeletal diseases among elderly. [15] It is noteworthy that the differences in trial characteristics were no longer significant after sensitivity analyses. However, these have limited power, as the proportion of trials specifically designed for elderly only reach 3, 2 and 0.1% respectively.

There are several limitations to this systematic review. Our database search was limited to papers published in the PubMed database. For feasibility reasons, we drew a random sample of 10% of the found publications and extracted a limited amount of trial characteristics from the publications. As a consequence, generalizability of our findings to all RCTs performed is limited. Further investigations would benefit from the extraction of more trial characteristics, such as upper age-related cut-off values for inclusion. We assume that the majority of RCTs maintain standard age-related cut-off values, i.e., 18 to 65 years. Further investigations could reveal whether the exclusion of older adults from participation in these RCTs is justified.However, the 1369 publications form a large database of RCTs conducted in 2012 enabling us to further elaborate associations between the enrolment of elderly and certain trial characteristics and to follow these associations over time.

In conclusion, there is a lack of RCTs specifically designed for older adults. This systematic review is the first to investigate the proportion of RCTs specifically designed for elderly across a wide range of disease categories and to compare a selection of trial characteristics of RCTs designed for elderly with those of RCTs not designed for older adults. Knowing that RCTs are still seen as a golden standard for the formulation of treatment guidelines, the underrepresentation of elderly in clinical trials has serious consequences for clinical practice. In the absence of evidence, treatment guidelines should not be automatically generalized to older patients. As older adults will increasingly form the majority of patients in medical practice, there is an urgent need for stronger evidence for the formulation of treatment guidelines specifically for elderly.

## Supporting Information

S1 PRISMA Checklist PRISMA checklist. (DOC)

S1 Appendix Included 1369 publications and trial characteristics. Includes the characteristics (title, authors, journal and trial characteristics) of the 1369 included publications.
(XLSX)
